# Enhancing Resident Doctors’ Preparedness and Confidence in Oncology Through a Practical Handbook

**DOI:** 10.7759/cureus.93085

**Published:** 2025-09-24

**Authors:** Haneefah Rubbani, Kyna Bailey, Mariallegra Gianfreda

**Affiliations:** 1 Oncology, Royal Stoke University Hospital, Stoke-on-Trent, GBR

**Keywords:** induction handbook, oncology, oncology handbook, quality improvement research, resident doctor induction

## Abstract

Aim

The aim of this quality improvement project (QIP) was to enhance the preparedness and confidence of resident doctors starting their oncology rotation at Royal Stoke University Hospital through the creation of a targeted, practical oncology handbook.

Methods

The QIP was designed using the Model for Improvement framework: Plan-Do-Study-Act (PDSA). A baseline survey was conducted among resident doctors during their oncology placement to assess their confidence in recognizing oncological emergencies, managing common oncological problems, and accessing resources to support them during the rotation. Feedback highlighted the need for a departmental oncology handbook covering these essential topics. The Oncology Handbook was subsequently developed and distributed in both print and digital formats. Its impact was evaluated through a post-intervention survey.

Results

Baseline survey responses from resident doctors (n = 5) revealed low confidence, with no respondents reporting being “fairly confident” or “very confident” in recognizing oncological emergencies or in investigating and managing any of the six surveyed oncology presentations. Confidence in locating resources was also low, with 40% reporting “not confident” and 40% reporting “somewhat confident.” Following the introduction of the Oncology Handbook, confidence improved substantially across all domains: 100% of respondents reported being either “very confident” or “fairly confident” in recognizing and managing core oncology presentations. In addition, 100% felt “very confident” about finding resources and guidelines for oncology, and 100% of respondents “strongly agreed” that the Oncology Handbook was useful. Qualitative feedback described the resource as “clear,” “practical,” and “invaluable during on-calls.”

Conclusions

A targeted, trainee-led intervention in the form of the Oncology Handbook significantly improved preparedness and confidence among resident doctors starting their oncology rotation. Integration into the formal induction program and establishing a process for regular updates are key next steps to sustain its impact.

## Introduction

Resident doctors rotating into oncology often face significant challenges adapting to a highly specialized environment due to unfamiliar terminology, complex treatment regimens, and oncological emergencies and presentations they may not have previously encountered. These demands can leave resident doctors feeling unprepared and anxious.

To address these challenges, the Oncology Handbook was developed at Royal Stoke University Hospital [1]. The handbook collates and consolidates essential information into a single practical resource, including management pathways for common oncology presentations, guidance on chemotherapy and immunotherapy complications, and key departmental information and contacts. Designed with accessibility in mind, it can be used on smartphones, computers, or as a physical reference in the oncology office, ensuring information is always readily available.

Inadequate induction processes can leave new residents insufficiently prepared at the start of their rotation with regard to their roles and responsibilities [2]. Evidence demonstrates that high-quality induction improves patient safety, minimizes service delays, and enhances the confidence of resident doctors [3,4]. Induction booklets and handbooks have also been shown to improve knowledge, confidence, and efficiency across various specialties [5,6]. For instance, Thomas et al. demonstrated that an induction handbook markedly increased resident doctors’ confidence in requesting appropriate investigations, orienting themselves to ward timetables, and understanding specialist terms [5]. Similarly, Dave et al. found that structured handbooks significantly improved both knowledge and confidence in new doctors managing complex surgical patients [6]. These findings suggest that specialty-focused resources are highly effective in reducing anxiety, improving role clarity, and supporting safer patient care.

Oncology-specific literature is limited, but available evidence suggests that resident doctors may experience difficulties when transitioning into cancer services, particularly regarding the management of systemic anti-cancer therapy-related toxicities [7]. A handbook as a learning resource can provide clear, concise, and practical guidance, which may be invaluable in supporting resident doctors’ knowledge and confidence in clinical practice [5,6].

## Materials and methods

Design and setting 

This quality improvement project was conducted in the oncology department at Royal Stoke University Hospital using the Plan-Do-Study-Act (PDSA) framework.

Data collection

Two anonymous online surveys were distributed in April 2025 to evaluate the effectiveness of the Oncology Handbook. The baseline pre-intervention survey, administered to resident doctors at the start of their rotation, explored satisfaction with induction, confidence in recognizing oncological emergencies, investigating and managing oncological presentations, ability to locate relevant resources and guidelines, and perceptions of the potential usefulness of a dedicated handbook.

The follow-up survey was distributed to the same cohort of resident doctors after implementation of the Oncology Handbook. It assessed the perceived usefulness of the handbook and its impact on residents’ ability to recognize oncological emergencies, confidence in investigating and managing common oncological presentations, and confidence in locating resources. Both surveys were created and analyzed using Google Forms (Google LLC, Mountain View, CA, USA). All responses were anonymized.

Target population 

Resident doctors rotating through the oncology department included Foundation Year 1 (FY1), Foundation Year 2 (FY2), Internal Medicine Training (IMT) residents, and Senior House Officers (SHOs).

Ethical considerations 

The project was reviewed and approved at the departmental level. As this was a service improvement initiative, formal ethics approval was not required. Participation in the surveys was voluntary and anonymous, and no conflicts of interest were identified.

## Results

A pre-intervention survey was conducted prior to the introduction of the Oncology Handbook, followed by a post-intervention survey after its implementation. Both surveys had five respondents: two FY1 doctors, two FY2 doctors, and one SHO. The respondents were the same individuals from the same cohort.

**Figure 1:**
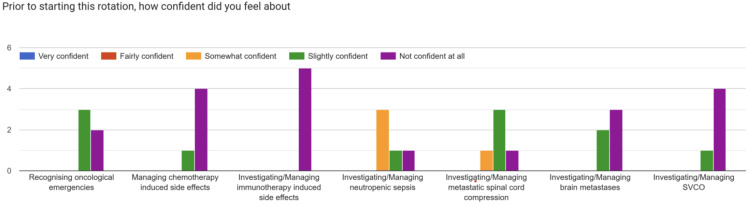
Confidence levels of resident doctors in recognizing oncological emergencies and in investigating and managing various oncology presentations prior to the introduction of the Oncology Handbook

The pre-intervention survey, distributed in April 2025 at the start of the oncology rotation (n = 5), asked resident doctors to rate their confidence in recognizing oncological emergencies and in investigating and managing core oncological presentations (Figure 1).

Overall, confidence levels were low across all domains. Most respondents reported being “not confident at all” or “slightly confident” in recognizing and managing oncological emergencies, chemotherapy-induced side effects, immunotherapy-related adverse events, spinal cord compression, brain metastases, and superior vena cava obstruction. Neutropenic sepsis was the only area where the majority reported feeling “somewhat confident,” though no respondents reported being “fairly confident” or “very confident.”

**Figure 2:**
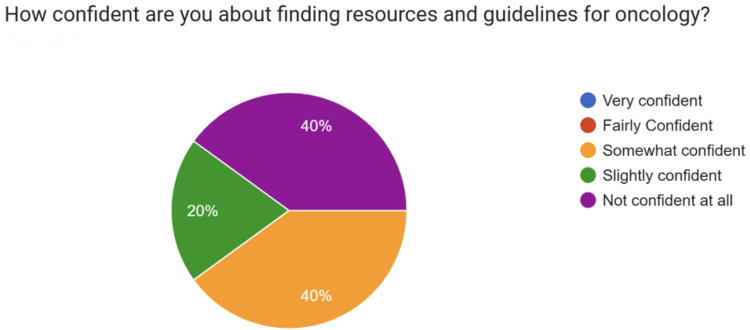
Confidence levels of resident doctors in locating resources and guidelines for oncology prior to the introduction of the Oncology Handbook

When asked about confidence in locating oncology resources and guidelines (Figure 2), 40% of respondents reported being “not confident at all,” 40% reported being “somewhat confident,” and 20% reported being “slightly confident.” None reported being “fairly confident” or “very confident.”

**Figure 3:**
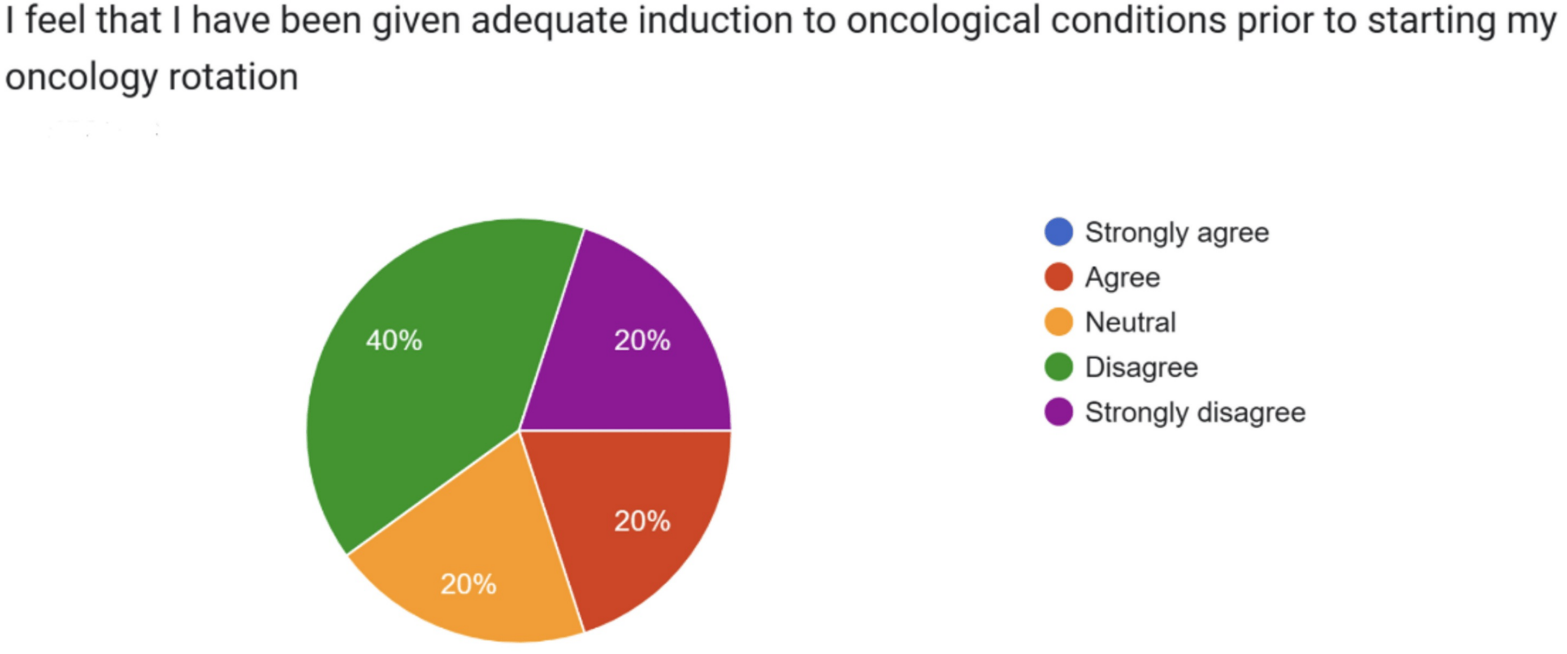
Resident doctors’ perceptions of receiving adequate induction to oncological conditions prior to starting their oncology rotation and prior to the introduction of the Oncology Handbook

When asked whether they felt adequately inducted to oncological conditions before starting the rotation (Figure 3), responses were evenly distributed: 20% selected “strongly agree,” 20% “agree,” 20% “neutral,” 20% “disagree,” and 20% “strongly disagree.” This spread reflects variability in perceptions of the quality and consistency of induction.

Taken together, these baseline findings demonstrate that new doctors entered oncology with limited confidence in investigating and managing common oncological presentations. This lack of confidence, compounded by inconsistent exposure to structured induction, underscores the need for a standardized and comprehensive induction resource.

**Figure 4:**
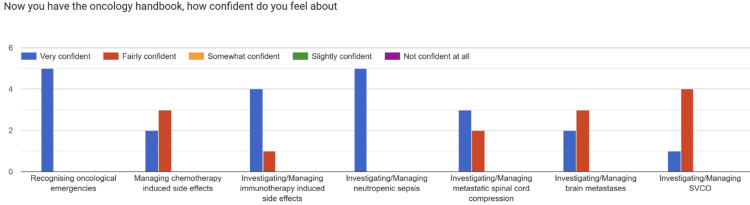
Confidence levels of resident doctors in recognizing oncological emergencies and in investigating and managing various oncology presentations following the introduction of the Oncology Handbook

Following the implementation of the Oncology Handbook, the post-intervention survey (April 2025) found substantial improvements across all domains. All respondents (100%) reported being “very confident” in recognizing oncological emergencies, and all reported being either “fairly confident” or “very confident” in investigating and managing core oncological presentations (Figure 4).

All respondents also reported being “very confident” in locating oncology resources and guidelines after the handbook’s implementation, and 100% “strongly agreed” that the Oncology Handbook was useful. Qualitative feedback described the resource as “clear,” “practical,” and “invaluable during on-calls.”

## Discussion

Prior to the introduction of the Oncology Handbook, induction for resident doctors consisted of a single day of short lectures, with most information conveyed verbally. As a result, residents were often uncertain about where to access resources, and perceptions of induction quality varied widely.

Following the introduction of the Oncology Handbook, resident doctors’ confidence and preparedness for their oncology rotation improved significantly. The intervention directly addressed gaps identified in the pre-intervention survey, including low confidence in recognizing oncological emergencies, investigating and managing core oncological presentations, difficulty in locating resources, and variability in induction experiences. These findings align with the literature. For example, Ahmed and Memon reported that an induction booklet for psychiatry residents led to clear and definite improvements in resident doctors’ experiences and satisfaction [8]. Similarly, Wickramanayake et al. observed increased confidence and knowledge among junior doctors in the ED following the introduction of an ED handbook at Queen Elizabeth Hospital, King’s Lynn, reflecting the improvements demonstrated in this study after implementation of the Oncology Handbook [9].

This project benefited from a robust survey methodology. Online surveys allowed secure data collection, straightforward analysis, and flexibility in case of errors. The use of pre- and post-intervention surveys enabled direct comparison of confidence levels, clearly demonstrating the handbook’s impact.

One limitation of this project is the small sample size, reflecting the limited number of residents rotating through oncology at Royal Stoke University Hospital. Furthermore, the quality of introductory lectures varied depending on the speaker, and handbooks in other institutions may differ, which could affect reproducibility. Although the surveys demonstrated marked improvements in confidence across all domains, the outcomes were based on self-reported confidence rather than objective measures of knowledge or clinical performance, introducing potential response bias. Finally, the long-term impact of the handbook was not assessed, leaving it uncertain whether confidence gains were sustained over time or translated into measurable improvements in clinical practice.

Despite these limitations, the project has several strengths. The intervention was resident-led, allowing it to closely reflect the needs of the target group, which likely contributed to its strong uptake and positive impact. The pre- and post-intervention surveys provided clear evidence of the handbook’s effectiveness. Another strength was its design: concise content, use of tables to present information clearly, and mobile-friendly formatting all enhanced accessibility and utility in daily clinical practice.

In summary, the Oncology Handbook has demonstrated that a professionally designed, specialty-specific induction tool can significantly improve preparedness and confidence among new oncology residents. Providing clear and concise guidance at the start of a rotation promotes safer practice, greater efficiency, and an improved resident experience.

## Conclusions

The Oncology Handbook resulted in a marked improvement in resident doctors’ confidence and preparedness for their oncology rotation. It effectively addressed gaps in induction and provided accessible, practical support for daily clinical practice. After its implementation, all participants reported increased confidence in recognizing oncological emergencies, investigating and managing oncological presentations, and locating relevant resources. Qualitative feedback highlighted its clarity and usefulness.

Incorporating the handbook into the formal induction program at Royal Stoke University Hospital, alongside a mechanism for regular review and updates, will help sustain its benefits. This practical, trainee-led intervention has the potential to enhance induction quality and contribute to safer patient care in oncology.

## Appendices

Pre-intervention survey

1. What training grade are you in currently?

a. Foundation Year 1

b. Foundation Year 2

c. IMT 1

d. IMT 2

e. SHO

2. Prior to your current oncology rotation how much time have you spent in oncology on placement or rotation?

(Free text)

3. Prior to starting this rotation, how confident did you feel about:

**Table 1:** Prior to starting this rotation, how confident did you feel about:

	Very confident	Fairly confident	Somewhat confident	Slightly confident	Not confident at all
Recognising oncological emergencies					
Managing chemotherapy induced side effects					
Investigating/Managing immunotherapy induced side effects					
Investigating/Managing neutropenic sepsis					
Investigating/Managing metastatic spinal cord compression					
Investigating/Managing brain metastases					
Investigating/Managing SVCO					

4. How confident are you about finding resources and guidelines for oncology?

a. Very confident

b. Fairly confident

c. Somewhat confident

d. Slightly confident

e. Not confident at all

5. I feel that I have been given adequate induction to oncological conditions prior to starting my oncology rotation:

a. Strongly agree

b. Agree

c. Neutral

d. Disagree

e. Strongly disagree

6. A departmental Oncology Handbook for resident doctors on common conditions and management of SACT sides effects would be useful

a. Strongly agree

b. Agree

c. Neutral

d. Disagree

e. Strongly disagree

7. Is there anything else that you would have liked to have received in your oncology induction to prepare you for investigating and managing common oncological scenarios?

(Free text)

Post-intervention survey

1. What training grade are you in currently?

a. Foundation Year 1

b. Foundation Year 2

c. IMT 1

d. IMT 2

e. SHO

2. Now you have the oncology handbook, how confident do you feel about:

**Table 2:** Now you have the oncology handbook, how confident do you feel about:

	Very confident	Fairly confident	Somewhat confident	Slightly confident	Not confident at all
Recognising oncological emergencies					
Managing chemotherapy induced side effects					
Investigating/Managing immunotherapy induced side effects					
Investigating/Managing neutropenic sepsis					
Investigating/Managing metastatic spinal cord compression					
Investigating/Managing brain metastases					
Investigating/Managing SVCO					

3. How confident are you post implementation of the Oncology Handbook about finding resources and guidelines for oncology?

a. Very confident

b. Fairly confident

c. Somewhat confident

d. Slightly confident

e. Not confident at all

4. I feel that the departmental Oncology Handbook for resident doctors on common conditions and management of SACT sides effects has been useful

a. Strongly agree

b. Agree

c. Neutral

d. Disagree

e. Strongly disagree

5. Is there anything else that you would have liked to have added to the oncology handbook to prepare you for investigating and managing common oncological scenarios?

(Free text)

6. Any further comments?

(Free text)
